# Improving breast cancer diagnosis by incorporating raw ultrasound parameters into machine learning

**DOI:** 10.1088/2632-2153/ac9bcc

**Published:** 2022-11-07

**Authors:** Jihye Baek, Avice M O’Connell, Kevin J Parker

**Affiliations:** 1 Department of Electrical and Computer Engineering, University of Rochester, Rochester, NY, United States of America; 2 Department of Imaging Sciences, University of Rochester Medical Center, Rochester, NY, United States of America

**Keywords:** breast cancer diagnosis, tissue characterization, machine learning, biophysical ultrasound feature, multiparametric analysis, multiparametric imaging

## Abstract

The improved diagnostic accuracy of ultrasound breast examinations remains an important goal. In this study, we propose a biophysical feature-based machine learning method for breast cancer detection to improve the performance beyond a benchmark deep learning algorithm and to furthermore provide a color overlay visual map of the probability of malignancy within a lesion. This overall framework is termed disease-specific imaging. Previously, 150 breast lesions were segmented and classified utilizing a modified fully convolutional network and a modified GoogLeNet, respectively. In this study multiparametric analysis was performed within the contoured lesions. Features were extracted from ultrasound radiofrequency, envelope, and log-compressed data based on biophysical and morphological models. The support vector machine with a Gaussian kernel constructed a nonlinear hyperplane, and we calculated the distance between the hyperplane and each feature’s data point in multiparametric space. The distance can quantitatively assess a lesion and suggest the probability of malignancy that is color-coded and overlaid onto B-mode images. Training and evaluation were performed on *in vivo* patient data. The overall accuracy for the most common types and sizes of breast lesions in our study exceeded 98.0% for classification and 0.98 for an area under the receiver operating characteristic curve, which is more precise than the performance of radiologists and a deep learning system. Further, the correlation between the probability and Breast Imaging Reporting and Data System enables a quantitative guideline to predict breast cancer. Therefore, we anticipate that the proposed framework can help radiologists achieve more accurate and convenient breast cancer classification and detection.

## 1. Introduction

There are a growing number of approaches that incorporate machine learning into the diagnosis of breast cancer using ultrasound and other imaging modalities (Sadoughi *et al* 2018, Houssami *et al* 2019, Houssein *et al* 2021, Lei *et al* 2021). Within these approaches, ultrasound imaging has several advantages. Ultrasound is relatively affordable with a lower price point among the portable units. This makes ultrasound a widely accessible, non-ionizing method for imaging studies, including underserved populations in developing countries (Marini *et al* 2019, Toscano *et al* 2021, 2022). Breast ultrasound is also utilized as an adjunct to x-ray mammography in certain cases, particularly the dense breast. Given these advantages, an intensive effort has been made to improve breast ultrasound using computer-assisted analyses over recent decades. Earlier approaches incorporated lesions’ features such as size, shape, texture, and boundaries within clustering and classification systems or rule-based algorithms (Cheng *et al* 2010, Liu *et al* 2010, Shan *et al* 2012, Wu *et al* 2012).

In recent years, further developments in artificial intelligence (AI) have broadened the types of breast cancer analyses. Previous studies utilizing machine learning approaches, such as support vector machine (SVM) (Cai *et al* 2015, Prabusankarlal *et al* 2015, Wu *et al* 2015) and random forest (Abdel-Nasser *et al* 2017), have output breast classifications of benign or malignant. These typically include feature extraction and selection, and their performance and running time rely on the efficiency of this step (Cheng *et al* 2010). Thus, machine learning approaches for breasts traditionally extracted simple texture or morphological features from log-compressed B-mode images. However, how to optimize feature extraction and selection remains unclear yet critical for performance. To address this feature dependency on performance and time-consuming processes, deep learning algorithms (Yap *et al* 2017, Becker *et al* 2018, Kumar *et al* 2018, Byra *et al* 2019, Qi *et al* 2019, Xu *et al* 2019, Wang *et al* 2020) have been recently applied to breast cancer detection not only in classification but also in lesion segmentation. Deep learning can advantageously extract data-driven and self-optimized feature maps from input images, and thus feature detection and selection are unnecessary. However, many approaches require large training sets to produce accurate diagnostic classifications, and collecting a large number of patient ultrasound data is challenging (Cheng *et al* 2010, Liu *et al* 2019, Houssein *et al* 2021). Moreover, due to the computational complexity of deep learning, there are limitations to the size and type of ultrasound signal inputs. Specifically, data is commonly processed (including log-compression and speckle reduction) before input to the algorithms. The raw ultrasound signals are not utilized, which limits classification performance (the input has lost much information compared to the raw ultrasound signals). Therefore, new approaches which utilize the raw ultrasound signals while exploiting the advantages of deep learning and machine learning would be valuable.

To address the limitations on utilizing B-mode images for AI input, several recent studies have employed radiofrequency (RF) data for convolutional neural network (CNN) based classification of malignant and benign breast masses. Kim *et al* (2022) generated parametric maps using RF data for the deep learning input, reporting a higher performance than B-mode input. Byra *et al* (2022) utilized RF data as deep learning input and achieved higher area under the curve (AUC) than B-mode for breast lesion classification and segmentation. Similarly, Gare *et al* (2022) used RF data along with B-mode for CNN input for the diagnostic labeling of benign and malignant, resulting in higher AUC than only using B-mode. Those studies have demonstrated that utilizing RF data can improve breast lesion classification performance compared to B-mode image input. Moreover, Jarosik *et al* (2020) compared CNN inputs of RF and envelope data, resulting in higher performance of RF than envelope, and further the performance was compared with Nakagami parameter based classifier, demonstrating better classification for the RF data based CNN. However, this study only utilized one parameter for the comparison, while further improvements in breast lesion identification can utilize multiparametric analysis with deep learning approaches or machine learning classifiers. For example, a recent study (Taleghamar *et al* 2022) attempted a deep learning approach to predict breast treatment response using quantitative ultrasound (QUS) multiparametric maps, yielding results comparable with previous deep learning studies. Taleghamar *et al* extracted features from raw ultrasound signals (RF data) and added this information as ‘preprocessing’. The extracted QUS feature images were used as input of the deep CNN to predict responder or non-responder to therapy. In contrast to the deep learning approach, multiparametric analysis using machine learning classifiers was performed (Uniyal *et al* 2014). Uniyal *et al* extracted multiple features from RF data and then tested SVM and random forests classifiers, resulting in higher performance of SVM than random forests. Further, Uniyal *et al* generated malignancy probability maps for breast lesions.

In this study, to improve diagnosis, we utilize SVM based raw ultrasound signal-driven features along with B-mode-driven features as ‘postprocessing’ of deep learning. Furthermore, beyond the two outputs of benign or malignant, we also designed our approach to provide the probability of malignancy utilizing these features and machine learning: our features are grounded on biophysical models of ultrasound-tissue interactions (Baek *et al* 2020a, 2020b, 2020c, 2021b).

Our goal is to improve the diagnosis of breast lesions imaged using conventional ultrasound scanners. To do this, we combine a number of recently developed biophysics-based analyses of ultrasound echoes, specifically the H-scan analysis and the Burr analysis of speckle (Parker and Baek 2020, Parker and Poul 2020), along with additional morphological measures of the lesion. These are combined mathematically using principal component analysis (PCA) and are separated in a multiparametric space by the SVM. The results of this synthesis, applied to a curated data base of 157 breast scans, demonstrate higher AUC than has been achieved in earlier studies investigating entire breast lesion (Uniyal *et al* 2014, Cai *et al* 2015, Becker *et al* 2018, Byra *et al* 2019, Jarosik *et al* 2020), including multiparametric analysis and AI. In addition to this approach, the overall analysis enables a visual display of the multiparametric and classification analyses, whereby colored overlays on the breast lesion indicate the localized probability of malignancy. This method, known as disease-specific imaging (DSI), enables an immediate visual ‘gestalt’ that is grounded in quantitative analysis and SVM classification metrics.

## 2. Background and theory

### 2.1. Probability of malignancy and DSI for breast cancer

DSI is an imaging approach which utilizes a multiparametric analysis to classify disease types and visualize disease progression and severity. The DSI approach first utilizes SVM for classification of disease categories within a training set and then assesses disease severity in a patient using multiparametric measures within the classified, multidimensional space. Lastly, the disease types and progression levels are assigned unique colors and color intensities overlaid on B-mode images. The DSI framework has been applied to liver diseases, where the SVM can classify liver fibrosis, steatosis, cancers, and normal liver (Baek and Parker 2021a, 2021b). Once a liver disease category was specified, then its progression level was estimated utilizing the inner product operation within the multidimensional, multiparametric coordinate system.

**Figure 1.**
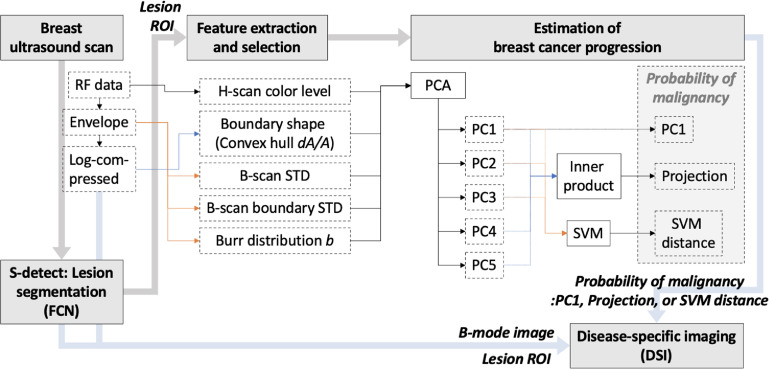
Flow chart for our breast cancer identification method including lesion detection, estimation of cancer progression, and imaging.

However, in the area of ultrasound breast exams, lesions are generally categorized as benign or malignant, and there are many sub-types within these categories. Some lesions within benign and malignant categories may have only slightly different multiparametric measures, and so the disease trajectories would have overlaps. In this paper, we consider these issues in breast cancer studies. We propose a modified DSI (figure 1), which skips the classification step of lesion sub-types but utilizes the SVM for disease progression quantification, including the classification of benign and malignant. We assigned a common color map for breast lesions, where color levels are distributed from green to red, indicating the likelihood of benign or malignant lesions, respectively. To enable this approach, the SVM builds a non-linear hyperplane classifying benign and malignant classes, and for any individual lesion the distance from the hyperplane is used for quantification of breast disease progression, indicating the probability of malignancy. The details of this approach are described in the next sections.

### 2.2. Quantification of breast cancer progression

Many parameters characterizing tissue signatures have been introduced, for example, several parameters extracted using ultrasound speckle, attenuation from beam propagation, shear wave attenuation and speed, and frequency domain analyses such as the H-scan (Baek *et al* 2020a, 2020b, 2021b, Basavarajappa *et al* 2021, Baek and Parker 2021b). Multiparametric analysis can be performed to combine more information from these numerous parameters while excluding their dependencies. PCA is a simple method to combine these parameters. The first principal component (PC1) tends to include the most efficient independent information from the parameters, and therefore in some cases PC1 by itself can be used for estimating disease progression. PC1 is a simple and fast approach to implement DSI for clinical ultrasound systems. However, some independent information is present in the second and higher principal components, and thus for more accurate estimation, a procedure including several principal components and then combining them into a 1D parameter may be advantageous.

As proposed in our previous work (Baek *et al* 2021a, Baek and Parker 2021b), the inner product resulting in projection can compare the similarity of multiple parameters to a reference set of measures associated with a particular disease. This assumes linear trajectories and simple disease models and was found to be effective in animal models. In this study, we generalize the concept for distinction between benign and malignant lesions without requiring unique labels of specific sub-types.

### 2.3. SVM classification distance

**Figure 2.**
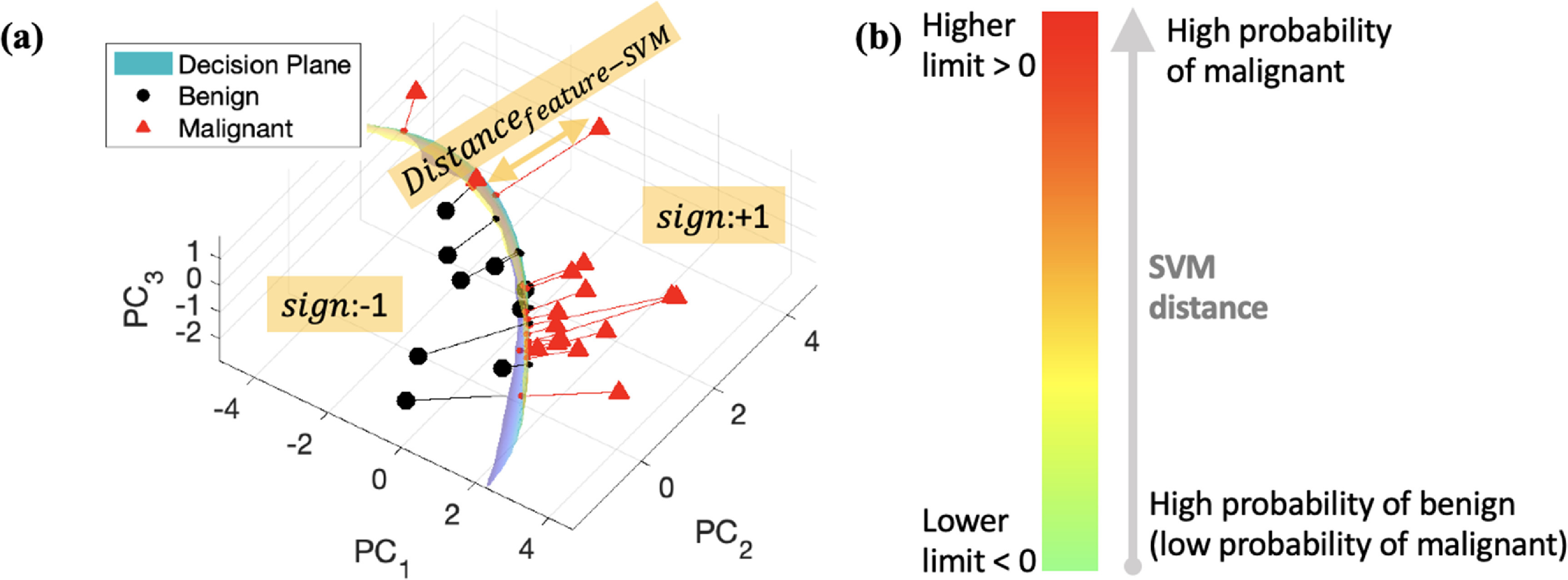
SVM distance and color map. (a) An example SVM hyperplane with measured features. The area classified as benign and malignant have sign −1 and +1, respectively. The distance between a feature and the plane is ${\text{Distanc}}{{\text{e}}_{{\text{feature}} - {\text{SVM}}}}$; an example distance is indicated by the orange arrow. (b) SVM distance mapping to a color map, representing probability of benign and malignant.

To construct a hyperplane for differentiation between benign and malignant, SVM training is performed with ground truth biopsy results. The use of a Gaussian kernel for the SVM allows us to develop a non-linear hyperplane. The box constant and kernel, as SVM parameters, are optimized to obtain a smooth and robust hyperplane, avoiding overfitting. More details regarding SVM parameter optimization can be found in (Baek *et al* 2020b). Once we define a non-linear hyperplane, we can calculate a distance between the plane and any data point in the multiparametric space (representing measurement from an individual lesion) to quantify disease progression, denoting the probability of malignancy, as illustrated in figure 2(a). This SVM distance is defined by:

\begin{equation*}{\text{sign}} \cdot {\text{Distanc}}{{\text{e}}_{{\text{feature}} - {\text{SVM}}}}\end{equation*}

where ${\text{Distanc}}{{\text{e}}_{{\text{feature}} - {\text{SVM}}}}$ is an absolute distance between a measured feature and the hyperplane, and sign denotes classification outputs −1 and +1 for benign and malignant, respectively. As shown in figure 2(b), we can set a lower and upper limit for the SVM distance, and the parameters have corresponding colors distributed from green to red, representing high probability of benign and malignant breast tissues, respectively. More details for the correlation between the color levels and probability are discussed in the section 4.3.

## 3. Methods

### 3.1. Study protocol

This study was approved by the Research Subjects Review Board at the University of Rochester and performed under the requirements of informed consent of. We enrolled 157 patients at the University of Rochester Medical Center, who had at least one suspicious breast lesion and were recommended to undergo biopsy or ultrasound imaging follow-up. The biopsy results were considered as ‘gold standard’. Patient characteristics are provided in table 1; among the 157 patients, seven patients were excluded because the study protocol was not followed correctly. An experienced radiologist categorized the breast lesions into three groups: (a) major types (seen frequently), (b) common, and (c) uncommon cases (infrequently seen or rare).

**Table 1.** Patient information.

Characteristic	Categories (count)/mean $ \pm $ standard deviation
Age	52.75 $ \pm $ 14.89
Ethic origin	White (*n* = 106), African American (*n* = 33), Asian (*n* = 3), Other categories (*n* = 8)
Lesion area size	0.87 $ \pm $ 1.09 cm^2^
Histology	Benign (*n* = 94), Malignant (*n* = 56)
Breast density	Almost entirely fatty (*n* = 9), Scattered areas of fibro glandular density (*n* = 41), Heterogeneously dense (*n* = 78), Extremely dense (*n* = 15), Density not available (*n* = 7)
List of major categories	Major benign (*n* = 68), Major malignant (*n* = 32), Common (*n* = 46, benign = 26/malignant = 20), Uncommon (*n* = 4)
Major categories: benign	Stromal fibrosis Fibroadenoma Fibro adenomatoid changes (FACs) Fibrocystic changes with stromal fibrosis Intraductal papilloma Cyst (microcyst cluster, ruptured cyst, simple cyst) Follow-up stable mass
Major categories: malignant	Ductal carcinoma *in situ* (DCIS) Invasive ductal carcinoma (IDC) Invasive lobular carcinoma (ILC) Invasive ductal and lobular carcinoma Invasive ductal carcinoma with micropapillary features Invasive mammary carcinoma (IMC)

Ultrasound examinations were performed using the RS85 ultrasound scanner (Samsung Medison Co., Ltd, South Korea) and a 3–12 MHz linear array transducer (L3-12A). A static image of the suspicious mass/lesion was acquired for each patient after the first screening of the lesion in the longitudinal and transverse planes. Among the 157 patients, only 154 patient scans were saved as RF data; for the others, only cine loops (without RF data) were saved, which cannot be analyzed for this study. The RF data were sent to Samsung Medison Co., Inc. (Seoul, South Korea), where they were converted into in-phase and quadrature (IQ) data (proprietary process for the RF data and its header information). From this set of data, we were able to reconstruct RF scan lines. However, only 121 patient scans which met the following consistent conditions were included in this study:

• Patients for whom the study protocol was followed correctly.
• A 9.4 MHz center frequency was used for transmission.
• Harmonic mode was not used.
• With Breast Imaging Reporting and Data System (BI-RADS) scores provided by radiologists.

The ultrasound breast images were reviewed by ten board-certified or board-eligible radiologists who have less than 5 years (*n* = 5) or over 10 years (*n* = 5) of experience. Thus, the only IQ data included were those where radiologists provided BI-RADS scores.

The BI-RADS scores provided by the ten radiologists were averaged using an area-preserving method of receiver operating characteristic (ROC) curve averaging (Chen and Samuelson 2014). The averaged BI-RADS score indicates the radiologists’ performance in breast diagnosis. Further, a deep learning framework to classify breast lesions, called S-detect in this paper, (S-Detect^TM^ for Breast in RS80A, Samsung Medison Co., Ltd, Seoul, Korea) (Han *et al* 2017, O’Connell *et al* 2021), was utilized, which indicated the performance of deep learning. More details are found in the section 3.2. S-detect classifies breast lesions as benign or malignant and outputs lesion boundaries. Lastly, our proposed analysis was applied to ultrasound breast images. The proposed method can combine multiple parameters, resulting in a quantified parameter indicating the probability of malignancy, and show a visual display called DSI. To define a breast lesion in an ultrasound image, we used the S-detect output of a lesion boundary. Also, we approximated (or smoothed) boundaries based on the S-detect results for efficient processing for parameter estimations. The S-detect boundary was used for estimating boundary shape measured using the convex hull estimation described in the section 3.3.2. The other parameters were not sensitive to boundary shapes, and thus approximated boundaries were used.

### 3.2. Breast lesion contour utilizing deep learning

Each imaged breast lesion was contoured by the S-detect after initialization by experienced radiologists in our previous study (O’Connell *et al* 2021). The S-detect was developed as a deep learning-based breast diagnosis system for ultrasound images, and it classifies breast lesions as benign or malignant.

In the S-detect approach, a breast lesion boundary is segmented using a modified fully convolutional network (FCN). Although the FCN (Long *et al* 2015) can automatically segment a lesion, S-detect modifies the FCN for semi-automatic segmentation to reduce errors due to unclear lesions on ultrasound images (Han *et al* 2017). Thus, the modified FCN requires clinicians to specify the center of a lesion and then it segments a boundary near the center. More details for the segmentation can be found in (Choi *et al* 2019). The next step is classification of breast tissues within the lesion boundary. GoogLeNet (Szegedy *et al* 2015) was modified by removing two auxiliary classifiers. Further, its input layer was modified to use gray scale ultrasound images rather than color input images, and it has two class outputs of benign or malignant rather than 1000 classes in GoogLeNet.

S-detect utilizes log-compressed envelope data as its input images. The input image is resized to a fixed pixel × pixel 2D image, and thus each image with a lesion has a different resize rate. Larger lesions have higher down-sampling rates than smaller lesions, resulting in more information loss for the larger lesions.

The S-detect was pretrained (Han *et al* 2017) and used in this study. The training was performed with 790 images acquired using the RS80A system (Samsung Medison Co., Ltd) and 6775 images acquired using other ultrasound machines (iU22, Philips Healthcare, Bothell, WA, USA; A30, Samsung Medison Co., Ltd). S-detect performance identifying breast cancer was reported in a previous study (O’Connell *et al* 2021).

### 3.3. Feature extraction—ultrasound parameters

We extracted features from ultrasound signals. Reflected ultrasound echoes are converted from RF data to IQ data, then envelope data, and then log-compressed data, in sequence. Deep learning approaches generally extract features from log-compressed data. However, to obtain higher accuracy based on multiparametric analysis rather than utilizing deep learning, we also extracted features from previous data, including RF data and envelope data, since the previous data have more information than the log-compressed data. For example, we can extract frequency information from RF data, but it cannot be obtained from envelope or log-compressed envelope data. Our selected features are presented in figure 1 with input data.

#### 3.3.1. H-scan color level

**Figure 3.**
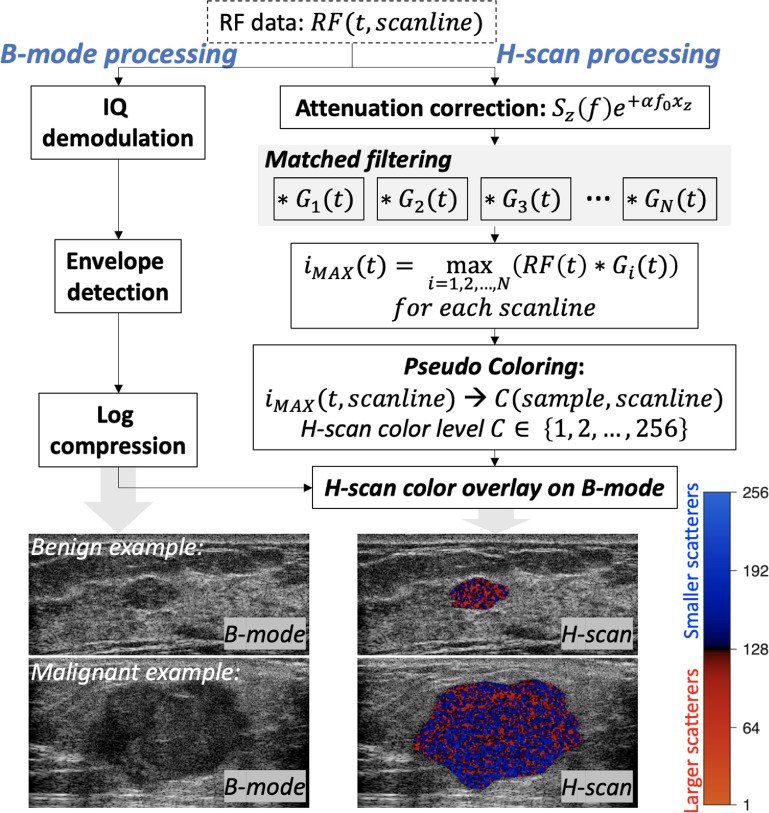
H-scan processing and example results of benign and malignant cases.

The H-scan is a matched filter analysis (Parker and Baek 2020), which enables the extraction of frequency information, including frequency spectrum distribution and shift, caused by attenuation and medium/scatterer changes (in size or connections between scatterers). H-scan processing and example results are shown in figure 3. The frequency at each time sample can be estimated by matched filters. This study used 256 Gaussian functions working as bandpass filters in the frequency domain; each Gaussian has its peak frequency from 5.2 MHz to 12.4 MHz with an equal frequency difference of 2.8 $ \times {10^{ - 1}}$ MHz. The filtering output using ultrasound RF data and the Gaussian filters resulted in 256 convolutional images. By selecting a maximum convolutional value at each time sample and each scanline, we can obtain the corresponding Gaussian index (${i_{{\text{MAX}}}}\left( t \right)$ in figure 3) with a peak frequency for the maximum. The Gaussian index, ranging from 1 to 256, becomes a color level ($C \in $ {1, 2, …, 256} in figure 3); the lower color levels indicate lower frequency components, while the higher levels indicate higher frequency components. The lower and higher components can denote larger and smaller scatterers, respectively. The lower to higher color levels are mapped into the red to blue colors of the H-scan; the H-scan color bar is shown in figure 3. In this breast study, the red to blue colors represent lower to higher probability of malignancy (higher and lower probability of benign), respectively.

Ultrasound propagation along depth causes attenuation, resulting in a red-shift of H-scan colors over depth. To compensate for this attenuation effect, attenuation-corrected RF data were used as inputs of the H-scan. For this correction (Parker and Baek 2020), we applied ${e^{\alpha {f_0}{x_z}}}$ to frequency spectrum ${S_z}\left( \,f \,\right)$ of RF data divided by ten zones over depth, where $\alpha $ is the attenuation coefficient, ${f_0}$ is the center frequency, ${x_z}$ is the average depth of each zone $z$ from 1 to 10, and ${S_z}\left( \,f \,\right)$ is the frequency spectrum of zone $z$. We used ${f_0}$ = 9.4 MHz and $\alpha $ = 1 dB MHz^−1^ cm^−1^ for our breast data. The measured color levels within a lesion were used as an H-scan parameter ranging from 1 to 256, called the ‘H-scan color level’.

#### 3.3.2. Boundary shape measured using the convex hull estimation [dA/A]

**Figure 4.**
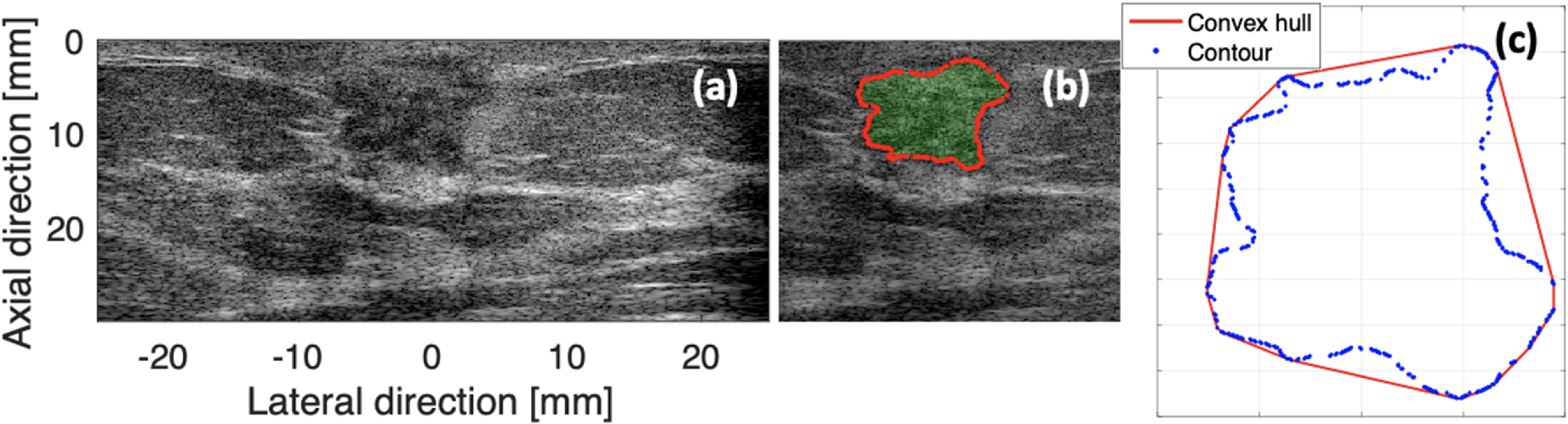
Boundary shape measured by convex hull. (a) An example breast scan. (b) Contoured lesion using a deep learning approach. (c) Convex hull of the lesion. Using the area within the contour (*A*) and the area difference between the contour and convex hulls (*dA*), boundary shape roughness of a breast lesion (*dA/A*) is measured.

Breast margin shape is one of the most critical features in BI-RADS to classify breast lesions as benign or malignant, since benign lesions generally show smoother boundary shape than malignant lesions. In previous research, morphologic features were introduced and utilized to delineate breast lesion boundaries: ellipse fit, convex hull, concave hull, elliptic-normalized skeleton, overlap ratio, solidity, and roundness (Chen *et al* 2003, Chang *et al* 2005, Alvarenga *et al* 2010, Cheng *et al* 2010, Wu *et al* 2012, Flores *et al* 2015). Flores *et al* reported higher performing morphological features among 26 features, and Chang *et al* also provided performance comparison between employed features. Based on these two studies, area comparisons between a lesion contour and a smoother version can be obtained by ellipse fit, morphological closing/opening, or convex hull methods. In this study, we employed the convex hull due to its relatively lower computation time compared to morphological closing, opening, and ellipse fit. For example, we have tested ellipse fit. Finding an optimal fit after several trials of ellipse fit is more time-consuming than finding a unique convex hull for any contours, but the boundary estimation accuracy was comparable for both methods. Therefore, with the convex hull estimation, we proposed a calculation method to accurately and quickly measure boundary roughness based on the best performing features mentioned above: overlap ratio (Flores *et al* 2015) and solidity (Chang *et al* 2005). As shown in figure 4, the convex hull of a breast lesion was obtained, and we estimated the lesion roughness using:

\begin{equation*}\frac{{dA}}{A} = \frac{{\left( {{\text{Convex}}{} \;{\text{hull}}{} \;{\text{Area}}} \right) - \left( {{\text{Contoured}}{} \;{\text{Area}}} \right)}}{{\left( {{\text{Contoured}}{} {\text{Area}}} \right)}}{} \end{equation*}

where *dA* is the area difference between the convex hull area and the contoured area, and *A* is the contoured area of a lesion. The boundary shape was defined by the proportion of the area difference (*dA*) to the lesion size *A*. The smoother boundaries have smaller *dA*/*A*, and thus this feature can be used to estimate the boundary shape of breast lesions.

To calculate this feature, log-compressed envelope data were used as input ultrasound images, and lesion contour using S-detect was utilized as input of the boundary shape measurement.

#### 3.3.3. B-scan parameters

There are numerous texture features extracted from B-mode images for breast tissue classification (Uniyal *et al* 2014, Flores *et al* 2015), which measure echogenicity and heterogeneity. Uniyal *et al* reported that the best feature among investigated B-mode texture features is mean intensity within a lesion. Flores *et al* ranked feature performance, finding better performing features which include features related to heterogeneity, such as autocorrelation and autocovariance. In this study, to extract texture features, we simply calculated B-scan intensity and standard deviation (STD) using log-compressed envelope data within a lesion to detect mathematical patterns. These two are representative features extracted from B-mode images, measuring echogenicity and heterogeneity.

#### 3.3.4. B-scan boundary parameters

**Figure 5.**
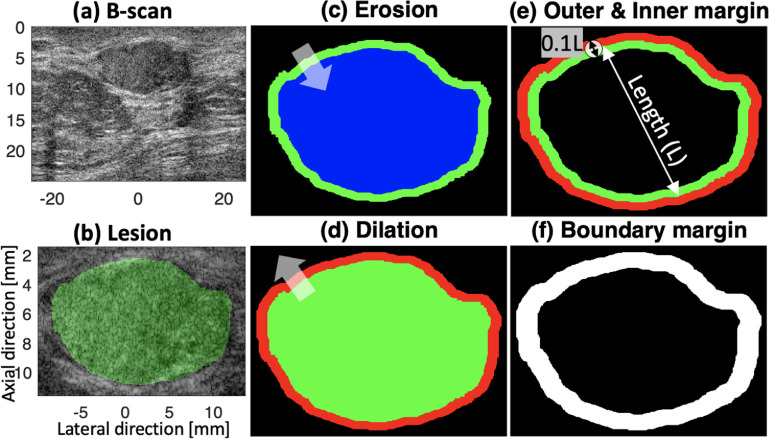
(a) An example B-scan image. (b) A lesion highlighted in green. (c) Morphological erosion was used to generate the blue area from the green lesion. (d) Morphological dilation was used to generate the red area from the green lesion. (e) The morphological erosion and dilation resulted in the inner and outer margins highlighted in green and red, respectively. (f) The margins in (e) were used as a boundary margin to calculate the feature ‘B-scan boundary standard deviation (STD)’.

In addition to extracting features within a lesion, we also detected a texture feature utilizing information near the lesion boundary. Characteristics of surrounding breast tissue are different than those of breast lesions and images from pathology and elastography often differentiate normal and abnormal tissue boundary regions (Barr 2019). Figure 5(a) shows an example breast image with a margin, where we can find the boundary between normal and abnormal tissues. Based on the boundary, the lesion was highlighted in green as shown in figure 5(b). Depending on the lesion type, the boundary textures may be more or less clear, and this boundary characteristic can help classify breast conditions. Therefore, we investigated tissues near the lesion boundary including inner and outer margins, as shown in figures 5(e) and (f), and within the margin, we extracted features using B-scan imaging: B-scan boundary intensity and STD. The inner and outer margins were defined using morphological erosion and dilation, respectively. Using the green lesion in figures 5(c) and (d), erosion and dilation generated the blue and red area, respectively. The green in (c) and red in (d) became the inner and outer margins of the lesion, respectively, which is shown in figure 5(e). Both margins combined are the boundary area as shown in figure 5(f). The morphological operations were performed with a disk shown in figure 5(e), and the disk size was determined so as to set the margin length at 10% of the lesion length. Then, within the margin in figure 5(f), we calculated B-scan intensity and STD.

#### 3.3.5. Burr power law parameters

We observed the histogram of ultrasound envelope data, and previous studies (Parker 2019a, 2019b) revealed that the histogram distribution of many soft tissues as a probability density function $P\left( A \right)$ is governed by the Burr distribution:

\begin{equation*}P\left( A \right) = \frac{{2A\left( {b - 1} \right)}}{{{\lambda ^2}{{\left[ {{{\left( {\frac{A}{\lambda }} \right)}^2} + 1} \right]}^b}}}\end{equation*}

where $A$ is an echo amplitude, and the distribution has two parameters: $\lambda $ (a scale factor that increases with amplitude and gain) and $b$ (a power law exponent dependent on scatterer distributions). Using an ultrasound envelope data, the curve fitting using the Burr distribution estimates the two parameters that can be used as features of our multiparametric analysis.

**Figure 6.**
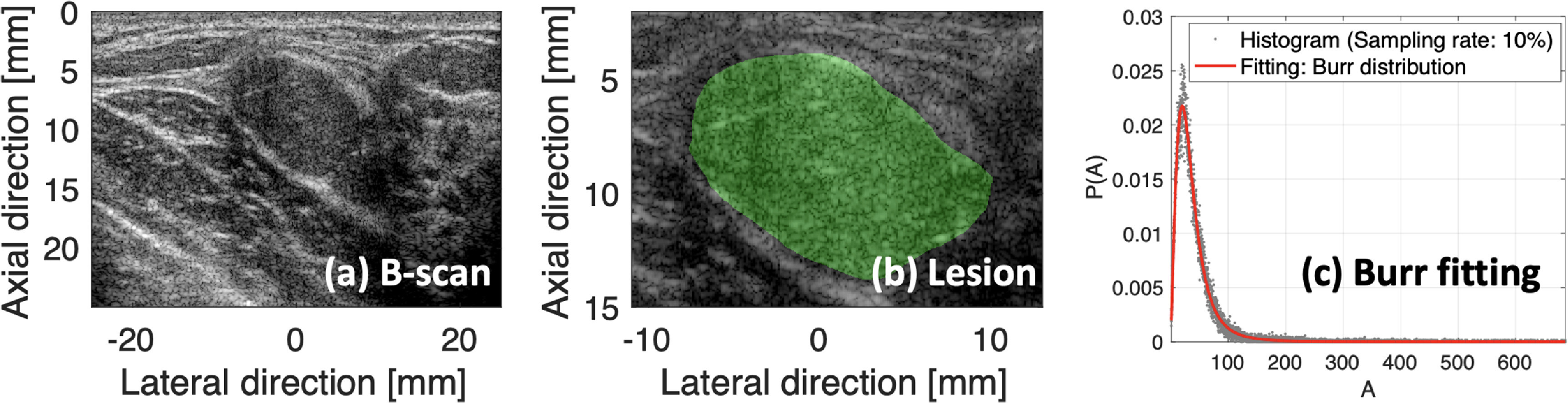
Burr distribution estimation of an example breast scan. Figures (a) and (b) show a B-mode image and a lesion in the image, respectively. (c) Example histogram plotted and Burr-fitted distribution.

For histogram estimation, one practical matter is the optimal setting of the histogram bin number as a sampling rate of the echo amplitudes distributed as a continuous real number. The sampling rate can be coarse, but it should be able to describe specific shapes of histogram distributions. Further, it should be sufficient enough to provide information enabling the curve fitting. However, an optimal sampling rate for each ultrasound frame can be different, and therefore we calculated the *R*-squared (*R* ^2^) for the Burr curve fitting within the sampling rates between 2% and 40% in equal intervals of 2%; the observed percentages are 2%, 4%, 6%, …, 38%, and 40%. Figure 6(a) shows an example B-scan, and figure 6(b) shows a lesion. Envelope data within the lesion were utilized for the curve fittings with different sampling rates. However, the estimated $\lambda $ and $b$ tended not to vary with sampling rate; the standard deviations of the $\lambda $ and $b$ estimates are 0.0287 and 0.0015, respectively. *R* ^2^ tended to decrease with increasing sampling rate. Therefore, we selected the sampling rate of 10% to have *R* ^2^ greater than 0.96 and to include enough bin numbers. We also considered the histogram bin numbers for small lesions. The minimum bin number for the 10% sampling rate is 229, however the minimum bin number for the 2% rate is only 46, which may be insufficient for fitting. In conclusion, the sampling rate of 10% was found to be optimal for all patient data, and was used to find optimal Burr parameters $\lambda $ and $b$.

### 3.4. Feature selection

As mentioned in the previous section 3.3, we estimated the following ultrasound parameters: H-scan color level and STD, boundary shape, B-scan intensity and STD, B-scan boundary intensity and STD, and Burr $\lambda $ and $b$. From these nine parameters, we selected five: H-scan color level, boundary shape, B-scan STD, B-scan boundary STD, and Burr *b*. For feature selection, AUC scores were compared for different parameter combinations using all 121 patient scans. ROC curves were obtained using (a) the ground truth biopsy results (benign or malignant), and (b) the performance observed from each parameter combination, which was measured by PC1 and SVM distance as the probability of malignancy. The AUC for the selected five-parameter combination was the highest compared to any other parameter combination, and therefore those features were selected for our analysis. The H-scan color level is a local parameter, whereas the others are global parameters.

### 3.5. Evaluation metrics

To evaluate each feature’s ability to differentiate between benign and malignant, one-way analysis of variance (one-way analysis of variance (ANOVA)) was used to compare the features for benign and malignant groups, calculating a *p*-value. According to an obtained *p*-value, the following statistical notations will be provided: ns (no significance) *p* > 0.05; * *p* < 0.05; ** *p* < 0.01; *** *p* < 0.001; and **** *p* < 0.0001.

To assess the accuracy of our proposed methods, we utilized the following evaluation metrics: (a) AUC, accuracy, sensitivity, and specificity from ROC curves, and (b) classification accuracy using the SVM. The metrics were obtained for our quantified outputs of PC1, projection, and SVM distance. Further, these outputs were compared with doctors’ BI-RADS scores to compare our results with doctors’ diagnostic performance.

To investigate lesion size dependance, we evaluated the performance of smaller to larger lesions, where lesion size thresholds of 0 cm^2^, 0.1 cm^2^, …, 0.9 cm^2^, 1.0 cm^2^ were observed. To be specific, for a threshold *A* cm^2^, only breast lesions greater than *A* cm^2^ were included for performance evaluations.

Moreover, we investigated the performance for all cases and for major benign and malignant. We first grouped the enrolled patients into (a) major benign and malignant, (b) common, and (c) uncommon cases. The ‘all’ case group includes all enrolled patients, but major benign/malignant only includes major benign and malignant cases, excluding the common and uncommon cases.

DSI provides qualitative and quantitative results, which are the imaging outputs and probability of malignancy (suggested by PC1, projection, or SVM distance), respectively. The DSI requires training for breast condition prediction. Therefore, we divided the enrolled cases into training and testing sets, and the previously mentioned evaluation metrics for the testing set, in addition to the training set, were observed to examine the potential prediction precision.

### 3.6. Training and testing data sets

Since the total number of lesions included in this study (*n* = 121 following the criteria in section 3.1) is insufficient to divide into training and testing sets, we generated five combinations of training/testing sets. Each training and testing set was randomly divided; the training and testing sets include 70% and 30% of data, respectively. This random generation was repeated five times, resulting in five randomly generated training/testing sets. Our results including classification accuracy were averaged over the five sets.

## 4. Results

### 4.1. Features and contributions

**Figure 7.**
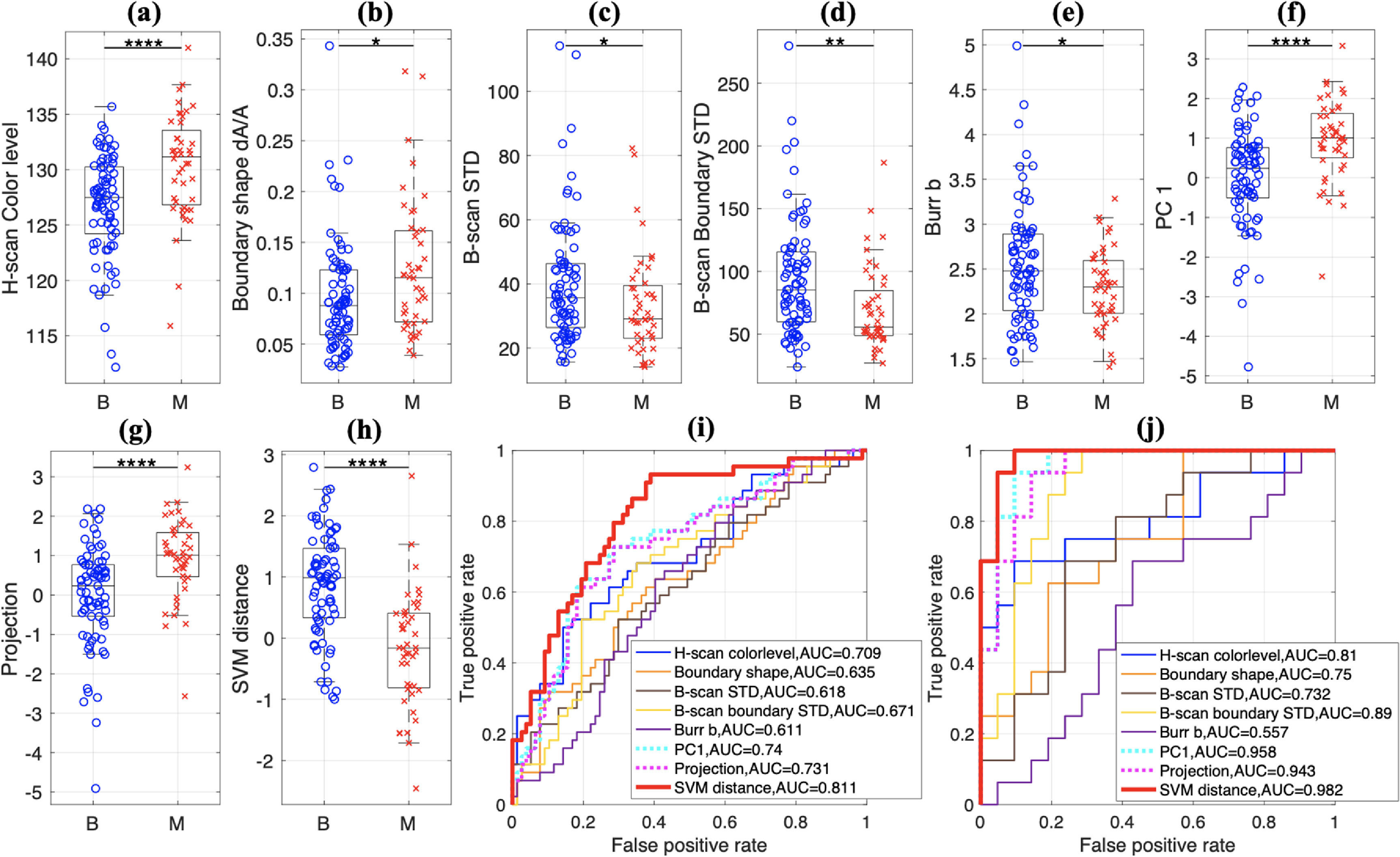
Performance of each feature (a)–(e) and combined features (f)–(h): all categories were included. (i) ROC curves for all categories. (j) ROC curves for major categories with size >0.4 cm^2^. The following notations are used for the statistics: ns (no significance) *p* > 0.05; * *p* < 0.05; ** *p* < 0.01; *** *p* < 0.001; and **** *p* < 0.0001. *GT: ground truth obtained from biopsy: B = benign and M = malignant.

The five features of H-scan color level, boundary shape, B-scan STD, B-scan boundary STD, and Burr *b* were selected since this combination resulted in the highest AUC compared to other parameter combinations. To obtain the AUC, we plotted ROC curves using PC1/projection/SVM distance and ground truths of benign and malignant. To assess whether the selected individual parameters can differentiate between benign and malignant, figures 7(a)–(e) show *p*-values for each feature. Figures 7(f)–(h) show *p*-values for the combined parameters of PC1, projection, and SVM distance. It is revealed that each feature can differentiate benign and malignant, showing *p*-values less than 0.05, however the combined parameters tend to result in lower *p*-values than individual features, meaning combining information from the five features provided better separation between benign and malignant. Figure 7(i) shows ROC curves for the five features and the three combined parameters, and table 2 provides AUC values for the ROC curves. The five features showed relatively comparable AUC, but the three combined parameters resulted in higher AUC than each feature. Moreover, SVM distance resulted in the highest AUC, showing the best performance in separating the benign and malignant cases. Note that figure 7 includes all enrolled patient categories of major benign/malignant, common, and uncommon. AUC scores are presented in table 2. The ‘All’ column includes all categories. The ‘Major’ (benign and malignant) columns only included major cases after excluding common/uncommon to calculate the AUC scores. Investigating only major categories with lesion sizes greater than 0.4 cm^2^ resulted in the highest AUC scores.

**Table 2.** All cases, all categories, and all lesion sizes were included to obtain the AUC scores in ‘All’. Further, after excluding common/uncommon cases, only major benign/malignant cases were utilized to obtain the AUC scores in the right two columns with all lesion sizes and lesion sizes >0.4 cm^2^. However, for further analysis, specific sizes were also considered, which enables higher AUCs as will be shown in the following sections. 95% confidence interval (CI 95%) for each feature is reported.

		AUC		
		All^a^ (*A* > 0 cm^2^) [CI 95%]	Major^b^ (*A* > 0 cm^2^) [CI 95%]	Major^b^ (*A* > 0.4 cm^2^) [CI 95%]
Individual feature	H-scan color level	0.71[0.61, 0.81]	0.75 [0.62, 0.87]	0.81 [0.66, 0.96]
	Boundary shape using convex hull *dA*/*A*	0.63 [0.53, 0.74]	0.70 [0.56, 0.83]	0.75 [0.59, 0.91]
	B-scan STD	0.62 [0.51, 0.72]	0.65 [0.51, 0.79]	0.73 [0.56, 0.90]
	B-scan boundary STD	0.67 [0.57, 0.77]	0.75 [0.62, 0.88]	0.89 [0.77, 1.0]
	Burr *b*	0.61 [0.51, 0.72]	0.61 [0.47, 0.76]	0.56 [0.37, 0.75]
Combined feature	PC1	0.74 [0.64, 0.84]	0.87 [0.77, 0.97]	0.96 [0.89, 1.0]
	Projection	0.73 [0.63, 0.83]	0.86 [0.76, 0.96]	0.94 [0.86, 1.0]
	SVM distance	0.81 [0.73, 0.90]	0.87 [0.77, 0.97]	0.98 [0.93, 1.0]
Radiologist BI-RADS score reading		0.78 [0.68, 0.87]	0.84 [0.73, 0.95]	0.92 [0.82, 1.0]
S-detect classification		0.76 [0.67, 0.85]	0.84 [0.73, 0.95]	0.84 [0.71, 0.98]

^a^ All: all lesion categories.

^b^ Major: major lesion categories as designated in table 1.

**Figure 8.**
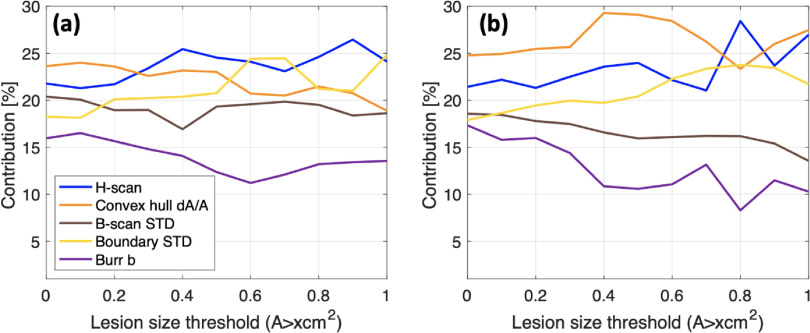
Contribution from each feature. (a) Includes all categories of major benign/malignant (B/M), common, uncommon cases. (b) Includes only major benign and malignant cases.

Our three combined parameters showed AUC greater than 0.86. These results demonstrate that we successfully selected features to combine information from each feature and to suggest a single combined parameter, and the best way to combine the parameters is the SVM distance. To investigate each feature’s contribution for the combination, we utilized PCA, which is shown in figure 8. Using the five features, PCA was applied to obtain the principal components, and the weight factors to calculate principal components from each feature were summed and used to calculate their contribution in figure 8. The contributions for all cases and major categories are shown. H-scan and convex hull parameters tend to show higher contribution than the others. Boundary shape tends to make the highest contribution for the smaller lesion sizes, whereas H-scan tends to make the highest contribution for the larger lesion sizes. Further, the contributions from B-scan STD and Burr *b*, which were based on ultrasound B-mode intensity, are likely to decrease as lesion sizes increase. As malignant lesions commonly have larger sizes and lower intensities, the hypoechoic area in larger sizes may provide less meaningful information than other features to distinguish tissue conditions, yielding less contribution than other features.

### 4.2. SVM classification and SVM distance

**Figure 9.**
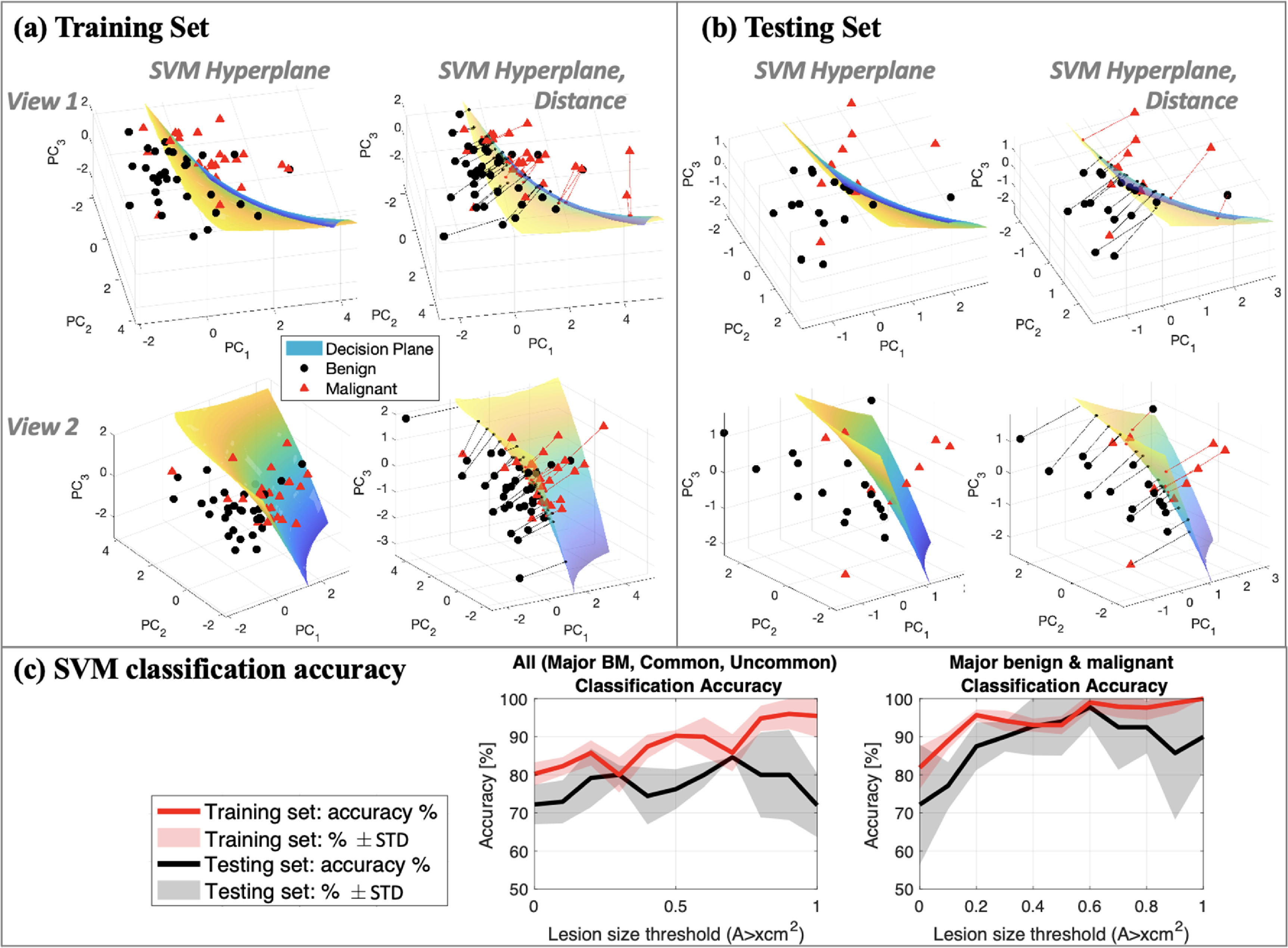
SVM hyperplane and SVM distance lines for (a) a training set and (b) a testing set. Two different views are provided. (c) SVM classification accuracy differentiating benign and malignant cases. The left plot includes all categories of major, common, and uncommon cases. The right plot only includes major benign and malignant cases.

The SVM classifier was used for calculating the SVM distance parameter and classifying benign and malignant cases. Figures 9(a) and (b) shows examples of SVM hyperplanes in two different views for training and testing sets. It also shows how the SVM distance was calculated by using the distance from each data point to the hyperplane, where a line corresponding to the distance is provided. Figure 9(c) shows classification accuracy for training and testing sets and also for all cases and major cases. The classification accuracy for major benign and malignant cases tends to be higher than all cases including major, common, and uncommon cases. The accuracy for the training sets is 80%–100%, and that for the testing sets is approximately 70%–80% for all cases and 80%–90% for major cases. The larger lesion sizes resulted in higher classification accuracies.

### 4.3. DSI performance

**Figure 10.**
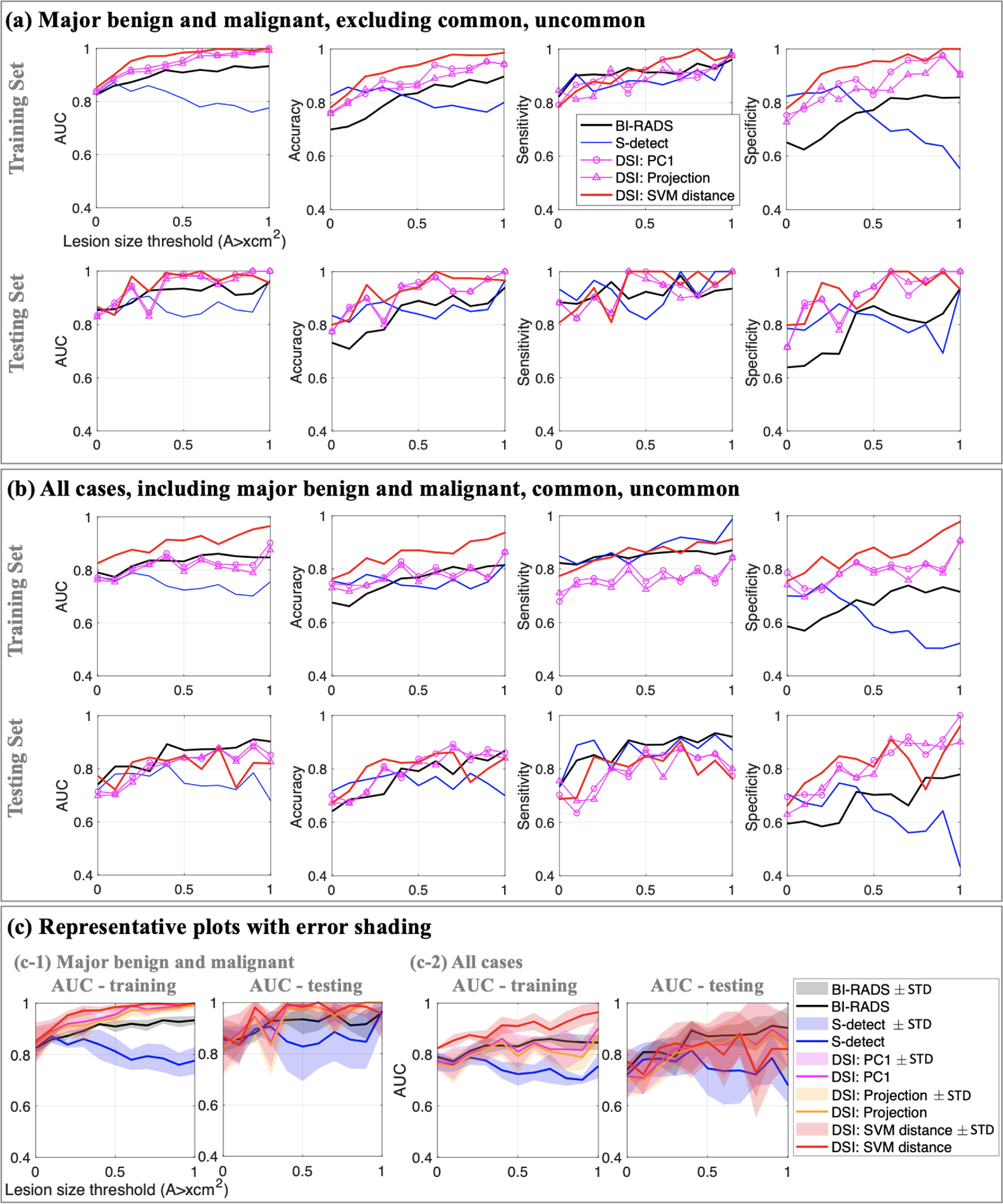
Performance comparison: AUC, accuracy, sensitivity, and specificity from DSI (PC1, projection, and SVM distance), BI-RADS score from radiologists, and S-detect output utilizing deep learning. The plots show quantification outputs of training and testing data sets. Figure (a) includes only major categories of benign and malignant. Figure (b) includes all categories of major, common, and uncommon cases. Figure (c) shows representative plots to show error bars using AUC scores. Measured average $ \pm $ STD area was shaded for each output. We generated five independent sets of testing/training data, so each data point is average for the five measurements. The other results of accuracy, sensitivity, and specificity showed comparable error range to that of AUC.

Figure 10 and table 3 show the quantitative outputs of AUC, accuracy, sensitivity, and specificity. We compared results from the radiologists, S-detect, and our quantification using PC1, projection, and SVM distance. The results were plotted for training/testing sets and for major and all categories in figure 10. Figures 10(a) and (b) include major categories of benign/malignant and all cases which includes major categories, common, and uncommon cases. For each plot, the performances were presented along with the lesion size thresholds ranging from 0 to 1 cm^2^, indicating lesion size dependency for breast tissue characterization. Each data point was obtained from the average of the five measurements since we generated five different sets of training and testing sets. Our quantification showed higher outputs (AUC, accuracy, sensitivity, and specificity) than the radiologists or S-detect. Further, among our three quantifications, the SVM distance parameter tends to show the best performance compared with PC1 and projection, but the results from PC1 and projection are still better than BI-RADS and S-detect. For smaller lesions, our results are slightly better or comparable to those of the radiologists and S-detect. However, in legion sizes greater than 0.5 cm^2^, the difference between our results and S-detect is increasing because the deep learning performance tends to decrease with increasing lesion size. Since S-detect resized the input images to the same pixel number, the images of larger lesions have higher down-sampling rates, which might cause information loss from the sampling process and thus lower the performance.

**Table 3.** AUC, accuracy, sensitivity, and specificity summaries for major and all categories with lesion sizes >0.5 cm^2^. Means and standard deviation (mean ± STD) of five training/testing sets are reported. The highest values were indicated using the bold font.

Lesion category	Dataset	Feature	AUC	Accuracy	Sensitivity	Specificity
Major^a^	Training	SVM distance	**0.984 ± 0.012**	**0.939 ± 0.039**	**0.922 ± 0.045**	0.955 ± 0.041
		BI-RADS	0.909 ± 0.023	0.835 ± 0.036	0.912 ± 0.019	0.772 ± 0.050
		S-detect	0.812 ± 0.055	0.809 ± 0.058	0.880 ± 0.092	0.744 ± 0.051
	Testing	SVM distance	**0.982 ± 0.024**	**0.940 ± 0.089**	**1.000 ± 0.000**	**0.903 ± 0.136**
		BI-RADS	0.935 ± 0.058	0.890 ± 0.082	0.924 ± 0.033	0.870 ± 0.109
		S-detect	0.828 ± 0.135	0.840 ± 0.134	0.820 ± 0.185	0.836 ± 0.157
All^b^	Training	SVM distance	**0.911 ± 0.023**	**0.870 ± 0.035**	**0.863 ± 0.051**	**0.881 ± 0.061**
		BI-RADS	0.832 ± 0.008	0.769 ± 0.010	0.856 ± 0.020	0.665 ± 0.012
		S-detect	0.724 ± 0.045	0.735 ± 0.048	0.861 ± 0.042	0.586 ± 0.075
	Testing	SVM distance	**0.849 ± 0.053**	**0.825 ± 0.052**	0.848 ± 0.109	**0.808 ± 0.055**
		BI-RADS	0.870 ± 0.017	0.791 ± 0.022	**0.889 ± 0.052**	0.703 ± 0.032
		S-detect	0.746 ± 0.104	0.738 ± 0.112	0.845 ± 0.108	0.646 ± 0.162

^a^ Major: major lesion categories.

^b^ All: all lesion categories as designated in table 1.

When comparing the results of the training and testing sets, we can conclude that our DSI training is not overfitted because the testing results are comparable to the training results with only slight differences, and most of the outputs (AUC, accuracy, sensitivity, and specificity) from the testing set are over 0.8.

Figure 10(c) provides representative plots with ±STD error ranges using AUC scores. Although there are slight overlaps between the parameters, the error bands are not completely overlapped, indicating meaningful differences between the different approaches. These plots can support that our DSI results are (a) better than or comparable to radiologists’ BI-RADS scores and (b) better than the S-detect. Figure 10(c) only provides plots for AUC scores, but the other metrics of accuracy, sensitivity, and specificity showed comparable error ranges with the AUC results. Additionally, table 3 reports quantitative measures with STD. To provide simple and easy to read plots, we have excluded the error shadings in figures 10(a) and (b).

**Figure 11.**
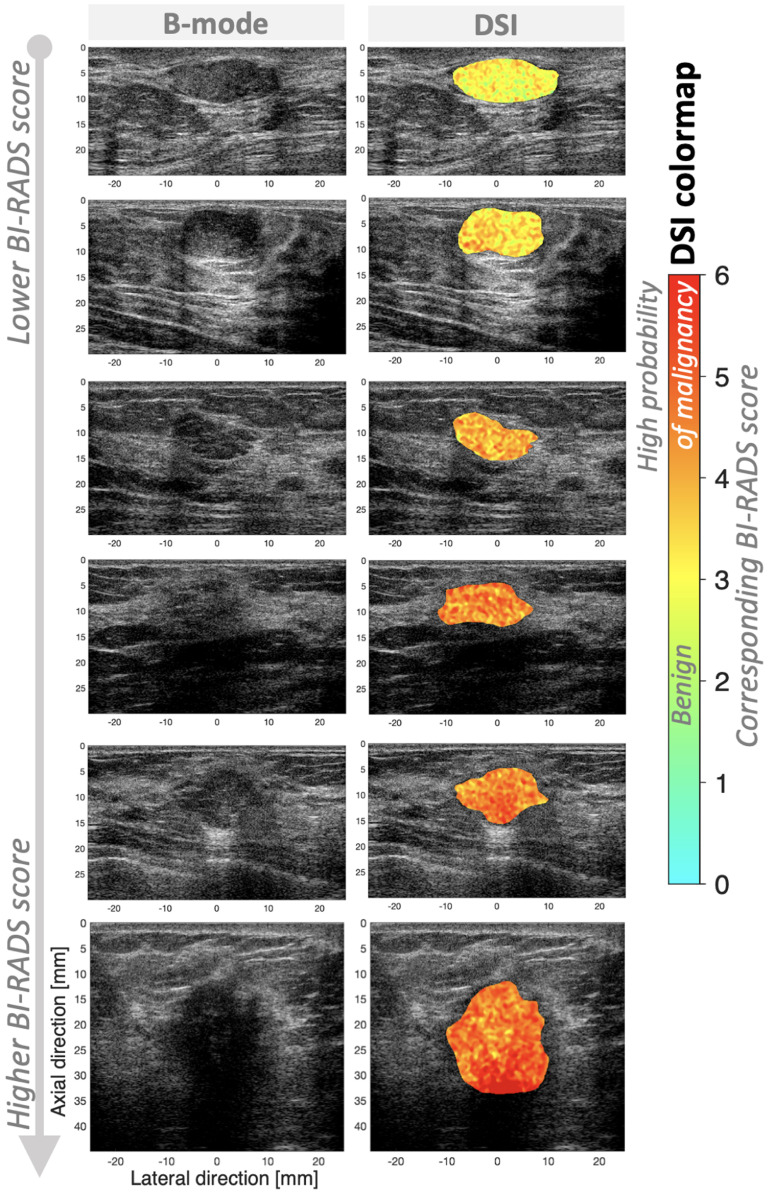
DSI and its color bar. On B-mode images, the localized probability of malignancy was color-coded using the given color map from light blue to red, indicating higher probability of benign and malignant, respectively. From top to bottom, the lesions were scored by the ten radiologists as BIRADS 3.20, 3.53, 3.90, 4.20, 4.47, and 4.93. For a quantitative guideline for DSI color levels and probability of malignancy, corresponding BI-RADS scores are provided. BI-RADS categories are defined by the American Cancer Society; 6: biopsy-proven malignancy, 5: highly suggestive of malignancy >95%, 4: suspicious for malignancy 2—94%, 3: probably benign, malignancy <2%, 2: benign, 1: negative, 0: incomplete.

Figure 11 shows representative DSI images. The six patient cases in figure 11 have lower to higher BI-RADS scores in order: 3.20, 3.53, 3.90, 4.20, 4.47, and 4.93; the BI-RADS were scored by ten radiologists and averaged. The localized probabilities of malignancy were estimated by PC1, projection, or SVM distance, which were encoded as colors between light blue to red, corresponding to benign to malignant, respectively, as provided by the color bar in figure 11. To obtain the images in figure 11, we applied trained DSI to major categories with lesion sizes greater than 0.4 cm^2^, and PC1 was used as color intensities. H-scan color level is the only localized parameter, which may differentiate tissue types within a lesion. As post-processing for DSI display, 2D median and Gaussian filters were applied to the measured combined parameters (color intensities) within a lesion.

As shown in figure 11, the lower BI-RADS score cases have a higher proportion of green or yellow colors, but the higher BI-RADS score cases have a higher proportion of red color components, which demonstrates that DSI can differentiate BI-RADS score differences.

**Figure 12.**
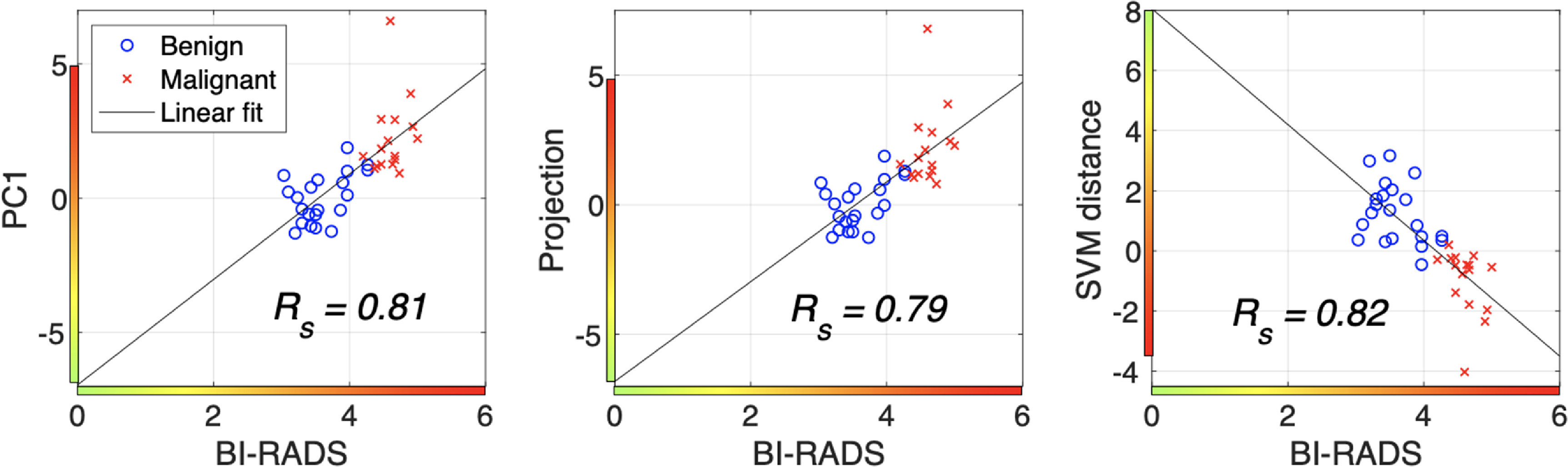
An example correlation between BI-RADS score and probability of malignancy. Corresponding DSI colors are provided on *x*- and *y*-axes.

The DSI colormap is provided in figure 11 with corresponding BI-RADS scores. The color intensities and hues of DSI visualize the probability of malignancy. Thus, to provide a quantitative guideline for breast cancer prediction, we suggest corresponding BI-RADS score for the DSI color levels (intensities) based on correlation between BI-RADS and the color levels. An example, mapping between BI-RADS and the color estimation is shown in figure 12, which was obtained using major categories with lesion sizes greater than 0.4 cm^2^, consistent with the example images in figure 11. Our DSI training was performed using ground truth biopsy results, and BI-RADS scoring was also evaluated using the ground truth biopsy. As shown in figure 10, DSI outperformed BI-RADS, however their correlation can be used as the guideline with Spearman’s correlation (*R_s_*) coefficients of 0.81, 0.79, and 0.82 for PC1, projection, and SVM distance, respectively.

## 5. Discussion

Our DSI framework is capable of producing testing set classifications with AUC, accuracy, sensitivity, and specificity between 0.95 and 1.0 for lesion sizes greater than 0.6 cm^2^ (0.9–1.0 for lesions greater than 0.4 cm^2^) for the major categories of benign and malignant lesions. Results are less accurate for smaller and non-major lesion types, possibly due to the paucity of data in these cases. Furthermore, the DSI analysis leads to a visual representation with color overlay indicating the severity of the disease from detailed multiparametric analysis. Additionally, the DSI color levels can suggest a quantitative guideline for the probability of malignancy.

These findings outperform previous research (Jarosik *et al* 2020, Byra *et al* 2022, Gare *et al* 2022) on utilizing RF data as AI input for breast classification, in which AUC were between 0.77 and 0.92. Moreover, there are studies (Uniyal *et al* 2014, Taleghamar *et al* 2022) utilizing RF data and extracting features for machine learning inputs with a similar approach as this study. Uniyal *et al* extracted features from RF ensembles or patches, used SVM for breast mass classification, and generated a malignancy probability map, of which AUC is 0.86; thus, our features improved breast lesion classification. Also, their malignancy probability map has RF patch-sized resolution, whereas our probability of malignancy map has a pixel-wise resolution.

Our training included only 121 patients, which may not be sufficient for comprehensive use in deep learning approaches, and thus we utilize the SVM classifier which advantageously requires less data compared with other deep learning and machine learning classifiers since it only utilizes support vectors near class boundaries (Bishop and Nasrabadi 2006). According to the properties of SVM, for feature dimension *m, m* + 1 support vectors are sufficient to determine SVM hyperplanes (Pontil and Verri 1998), but larger training sets help obtain more reliable results. Moreover, we optimized the SVM parameters to avoid overfitting. Hence, we have constructed more robust planes, meaning the parameter settings were selected to obtain smooth hyperplanes, although the planes still have higher-order curvature, as shown in figures 9(a) and (b). However, as seen in figure 9(c), when investigating all categories, the classification accuracy of the testing sets for lesion sizes greater than 0.8 cm^2^ might be slightly lower than a result from successful training, but it is still over 70%. This is because we included non-major categories and excluded smaller lesion sizes less than 0.8 cm^2^, and thus the enrolled patients are less than 121 for non-major categories. Further studies, including sufficient patient numbers, are required to enable our approach to be used by commercial ultrasound machines since this study have demonstrated the potential of quantifying the probability of malignancy with a limited data set.

Previous studies for breast cancer detection or classification utilizing machine learning were summarized in a survey paper (Cheng *et al* 2010). Since selecting useful features is crucial, previous studies tend to extract more features and then use several approaches to select some features (Kohavi and John 1997). However, most of the studies extract features from post-processed and log-compressed B-mode images, and thus there are limitations to extracting more information for increased performance. Specifically, a breast tumor diagnosis study (Chang *et al* 2005) extracted six features, including the aspect ratio between the maximum and minimum diameter, but only one feature showed a difference between benign and malignant. Another study (Wu *et al* 2012) extracted 30 features, but differently selected feature combinations resulted in similar classification accuracies. Therefore, instead of focusing on extracting more features from the log-compressed images as is the trend of previous studies, this study selected only five features, which can be considered independent and were extracted from RF data. We first showed that the five features can differentiate between benign and malignant (figure 7). The boundary shape and texture feature extracted from B-mode (B-mode STD and B-mode boundary STD) are commonly-used representative features for breast cancer detection. For B-mode texture features, we simply measured mean and STD within a lesion since this study focuses on extracting features from RF data; but it remains for future work whether selecting better performing features among numerous B-mode texture features, summarized in a previous work (Flores *et al* 2015), could improve performance of our proposed method. We tried to select minimum and independent features from previous studies. And then, we added two more independent features of H-scan and Burr parameters extracted from RF and envelope data, respectively; this group’s previous studies have also demonstrated that the two parameters are capable of distinguishing different disease types (Parker and Baek 2020, Baek *et al* 2020a, 2020b, 2021b, 2021c). A longer study of comparative models, including Nakagami and Homodyned K distribution, with Burr parameters would be quite interesting but is beyond the scope of this study. Meanwhile, we speculate that the relatively low performance of Burr *b* is influenced in this study by the many hypoechoic lesions in which the speckle is strongly influenced by the noise floor. As mentioned in section 3.3, RF and envelope data include more information than log-compressed data. Importantly, analyzing RF data enables the extraction of frequency-dependent information which cannot be found in log-compressed images. These newly implemented methods enable us to achieve higher performance with fewer features compared with the previous trend of using more features. Moreover, the careful selection of fewer features can reduce processing time and computational complexity. It can be noted that our final accuracy may be scanner dependent, because our analysis of RF signal information relies on the collection of relatively high bandwidth and signal-to-noise echoes. Furthermore, the B-scan related metrics benefit from high spatial resolution, so our overall results may not be achievable across all varieties of scanners, this remains for further investigation.

We demonstrated that our DSI framework yielded better performance than radiologists’ BI-RADS scores and the established deep learning approach of S-detect (figure 10). First, for the comparison with the BI-RADS scores, radiologists utilized B-mode images, and thus they tend to only rely on information on lesion boundary shapes and B-mode brightness. The post-processed B-mode images in ultrasound systems are optimized for the human eye, but in terms of signal processing, the images lose information while processing gray scale from RF channel data. Our DSI framework extracts features from summed RF data to utilize more information to characterize breast tissues, which cannot be seen by radiologists viewing only B-mode images. Secondly, for the comparison with S-detect, deep learning approaches generally utilize the post-processed B-mode images, reducing the computational complexity of utilizing raw data (such as RF) while reducing memory and processing time requirements. Ultrasound imaging applications in commercial ultrasound systems are sensitive to these issues. To address this within our DSI framework, we employed the SVM, which is less intensive in processing and therefore can utilize parameters obtained from RF data as its input. This approach allowed us to extract more information from ultrasound signals, which contributed DSI’s better performance compared to S-detect.

This study demonstrated that our DSI approach has the potential to be used in breast cancer diagnosis. However, to be used as an application in clinical ultrasound systems, further studies to make it faster would be advantageous. This study has focused on obtaining better performance, but to consider tradeoffs between performance and time, the following need to be addressed. (a) In feature extraction, for the H-scan parameter, we can use a smaller number of Gaussian-matched filters instead of the 256 filters used in this study. Reducing the filter number renders H-scan less precise but faster. (b) In the combined parameter for estimation of breast cancer progression, there are three parameters that have been considered as results of multiparametric analysis: PC1, projection, and SVM distance. According to our results, the SVM parameter performs the best, while PC1 and projection have comparable but slightly lower performance. However, comparing their processing times, PC1 is the fastest (projection is comparable, whereas SVM distance takes much more time than the others). Thus, utilizing PC1 as the color intensity of DSI may result in slight performance loss, but could be much faster. (c) S-detect does not work in real-time when used for lesion contour, and thus it outputs results onto a still frame. Our DSI utilizes the S-detect result of lesion boundary. Hence, the DSI would show results in a still frame, but efforts to make the overall boundary detection and DSI processing faster would be helpful for clinicians to see results in less time, including lesion identification and the color display.

## 6. Conclusion

We have developed a DSI framework for breast tissue characterization—including classification and prediction of the severity of breast cancer—utilizing multiparametric analysis. This study modified the previously proposed DSI classification of separate pathologies into a simpler classification of benign or malignant, with a corresponding color overlay map to visualize the results of the analysis. This newer version of DSI enables the quantitative analysis of human breast lesions, including more complicated classes and types. The approach produces an AUC above 0.98 for the most common types of lesions and sizes.

Our approach to combine multiparametric analysis with machine learning achieved better performance than both radiologists or the available deep learning system. Finally, we demonstrated that applying DSI not only allows for more accurate identification of breast lesions than deep learning alone, but also provides a visual display. Therefore, we anticipate that the DSI framework could be utilized to enable more accurate and rapid tissue characterization of human breast lesions in the clinical field.

## Data Availability

The data that support the findings of this study are available from the corresponding author upon reasonable request.
